# Formal water oxidation turnover frequencies from MIL-101(Cr) anchored Ru(bda) depend on oxidant concentration†
†Electronic supplementary information (ESI) available. See DOI: 10.1039/c8cc02300j


**DOI:** 10.1039/c8cc02300j

**Published:** 2018-06-12

**Authors:** Asamanjoy Bhunia, Ben A. Johnson, Joanna Czapla-Masztafiak, Jacinto Sá, Sascha Ott

**Affiliations:** a Department of Chemistry – Ångström Laboratory , Uppsala University , Box 523 , Uppsala 75120 , Sweden . Email: Sascha.Ott@kemi.uu.se; b Department of Chemistry , National Institute of Technology-Puducherry , Karaikal 609 609 , India; c Institute of Nuclear Physics Polish Academy of Sciences , Krakow PL-31342 , Poland; d Institute of Physical Chemistry , Polish Academy of Sciences , ul. Kasprzaka 44/52 , Warsaw 01-224 , Poland

## Abstract

The molecular water oxidation catalyst [Ru(bda)(L)_2_] has been incorporated into pyridine-decorated MIL-101(Cr) metal–organic frameworks. The resulting MIL-101@Ru materials exhibit turnover frequencies (TOFs) up to ten times higher compared to the homogenous reference. An unusual dependence of the formal TOFs on oxidant concentration is observed that ultimately arises from differing amounts of catalysts in the MOF crystals being active.

## Abstract 2

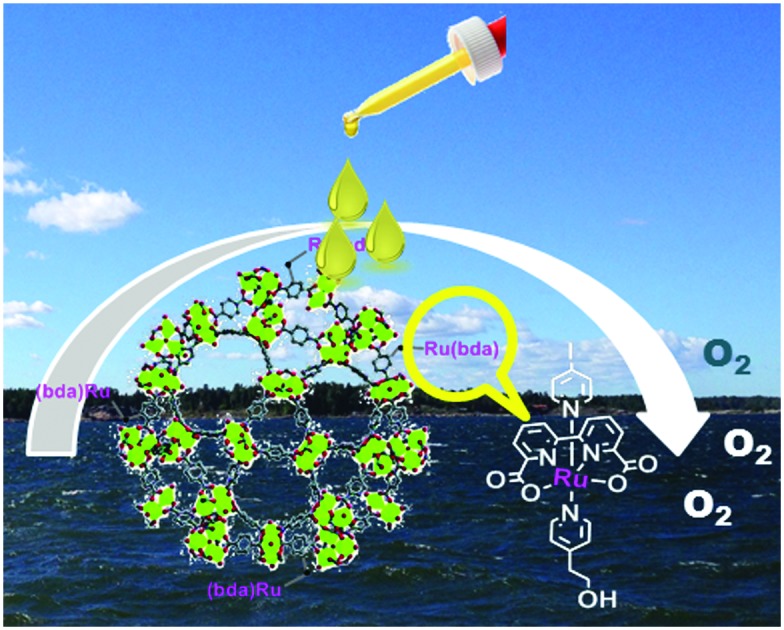
[Ru(bda)(L)_2_] incorporated into the MIL-101(Cr) metal–organic framework catalyzes water oxidation faster than a homogenous reference, with the number of active catalysts depending on oxidant concentration.

## 

Water oxidation is at the heart of artificial photosynthesis and central to future energy conversion schemes.^1^ Amongst molecular water oxidation catalysts, ruthenium-based complexes are particularly appealing because of their high catalytic activity.^2^ While most of these studies have been conducted in solution phase, it is generally desirable to incorporate molecular catalysts into heterogeneous supports. Ideally, this immobilization should not only bring practical advantages such as recyclability, but also stabilize the molecular integrity of the catalyst. Up to now, only a few works on immobilized Ru-based water oxidation catalysts have been reported, and most utilize surface modifications to electrodes.^3^ Recently, Li *et al.* introduced the well-known [Ru(bda)(L)_2_] catalyst (bda = 2,2′-bipyridine-6,6′-dicarboxylate) into the nanocage of mesoporous silica (SBA-16) by using a ship-in-a-bottle approach.^4^ In this construct, multiple catalysts are in close proximity within the same cage, creating a situation that promotes water oxidation by a binuclear radical (I2M) coupling mechanism.^4^ This pathway is thermodynamically preferred in [Ru(bda)(L)_2_]-type complexes compared to the alternative where water nucleophilically attacks (WNA) a high valent metal species.^5^ As the physical trapping in the silica pores is potentially not ideal from a leaching point of view, the design of systems where molecular catalysts are linked to the support by covalent or coordination bonds is desirable.

Metal–organic frameworks (MOFs) possess a unique combination of properties such as extremely high surface areas, open structures, tuneable pore size and functionality.^6^ As a result, MOF-based catalysis has attracted considerable attention over the past years, as it combines the benefits of heterogeneous and molecular catalysis.^7^ Among the many strategies for synthesizing catalytic MOFs, the incorporation of catalytically active species into pristine MOFs by post-synthetic modifications (PSM) has become a powerful approach.^8^ With an increasing number of MOF-based catalytic processes being reported, for example in Lewis acid-based catalysis,^9^ hydrogen production,^10^ CO_2_ reduction^11^ and many others, the oxygen evolution reaction (OER)^12^ is still underexplored. The challenges in MOF-catalysed OER are manifold, and include the limited stability of many MOFs in aqueous media at often extreme pH,^18^ and their reactivity towards strong oxidants. Moreover, the oxidants need to be able to access the molecular catalysts within the MOF crystals to engage all catalysts. Lin and coworker reported a family of UiO-type (UiO = University of Oslo)^13^ MOFs for water oxidation in which IrCp*Cl (Cp* = pentamethylcyclopentadienyl) was bonded directly to the pore surface of the MOFs containing bipyridine (bpy) and phenylpyridine linkers (Ir-bpy-MOF).^14^ Using Cerium(iv) ammonium nitrate (CAN) as oxidant, the initial TOF for Ir-bpy-MOF was about 0.12 min^–1^, which was about 40 times less than that of the corresponding homogeneous catalyst [IrCp*Cl(bpy)]Cl under similar conditions (*ca.* 3 mM CAN). This discrepancy is due to the hindered transport of CAN (molecular size ∼1 nm) into the bpy-MOF (pore size ∼1 nm) that renders many catalysts in the interior of the MOF inaccessible.^14^

To minimize these limitations, we chose a MOF with larger pore sizes for the incorporation of a Ru(bda)-based water oxidation catalyst in the present study. MIL-101(Cr) ([Cr_3_(μ_3_-O)(OH)(BDC)_3_(H_2_O)_2_]·∼25H_2_O; BDC = benzene-1,4-dicarboxylate) contains large inner cages of 2.9 and 3.4 nm diameters with pore aperture window diameters of 1.2 and 1.6 nm, respectively.^15^ The incorporation of Ru(bda) into MIL-101(Cr) was achieved using two different linking motifs, both of which use an axial pyridine ligand as anchoring point. This position was chosen as the pyridine–Ru coordination bond was recently shown in a crystallographic study of decomposition products to be the most stable one of the complex.^16^ Both materials, MIL-101-2@Ru and MIL-101-4@Ru were prepared using PSM strategies (Scheme 1, see ESI† for details).^8^

**Scheme 1:**
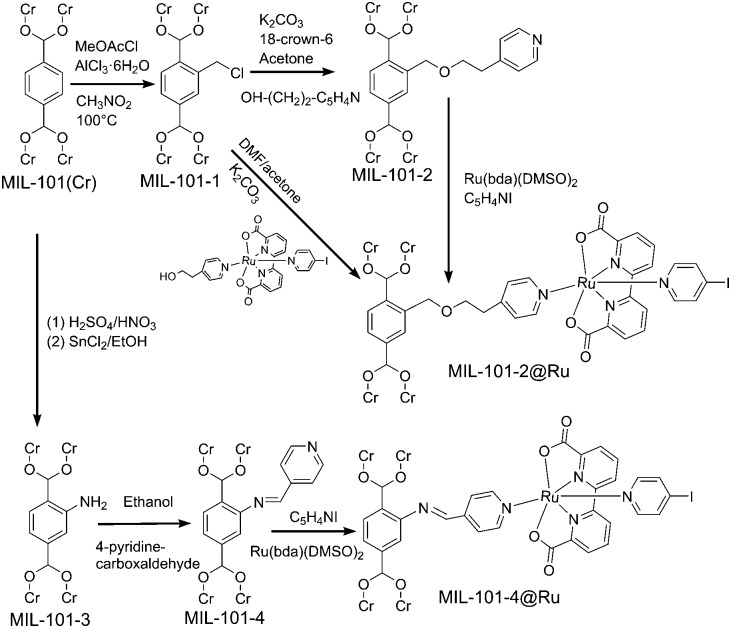
Schematic representation of the post-synthesis modification of MIL 101(Cr) *via* a three step or two step functionalization processes.

MIL-101-2@Ru and MIL-101-4@Ru differ in the linkage type, but also in catalyst loading. ICP-AES reveals that for MIL-101-2@Ru, 1.52% of the total BDC linkers where functionalized with a Ru(bda) unit, while catalyst loading in MIL-101-4@Ru is considerably higher at 8.15%. To gauge the distribution of Ru in MIL-101-4@Ru, the Cr : Ru ratio was determined by EDX spectroscopy to 5.8 : 1 (Fig. S1, ESI†). While EDX is a more surface sensitive technique, this value is very similar to that of ICP-AES (Cr : Ru ratio = 6.3 : 1), indicating that the Ru centres are statistically distributed in the framework. Further supporting this notion is the fact that such a high catalyst loading could not be accommodated exclusively at or near the surface due to simple space restrictions. For MIL-101-2@Ru, EDX studies were inconclusive due to poor signal-to-noise ratios.

Powder X-ray diffraction (PXRD), scanning electron microscopy (SEM), Ru K-edge X-ray absorption spectroscopy (XAS), nitrogen physisorption analysis, and other spectroscopic techniques confirm the structural integrity of the materials. It is important to stress that the characteristic peak positions and relative intensities in the PXRD spectra of all MIL-101(Cr) throughout all PSM processes are well preserved without any observable evidence for decomposition (Fig. S2 and S3, ESI†). This further indicates that the basic 3D framework is retained, and thus the pentagonal (2.9 nm) and hexagonal (3.4 nm) channels are accessible to reactants. SEM images of all materials show identical cubic micro-crystals (Fig. S4, ESI†), proving that the overall morphology is unchanged during the PSM reactions. As expected, the BET surface areas of the materials decrease noticeably upon functionalization and catalyst loading (Fig. S5 and S6, ESI†). The final materials MIL-101-2@Ru and MIL-101-4@Ru feature surface areas of 1634 ± 100 m^2^ g^–1^ and 1413 ± 100 m^2^ g^–1^, respectively, compared to that of the non-functionalized MIL-101(Cr) (3160 ± 100 m^2^ g^–1^). The molecular integrity of the catalyst within the MIL-101(Cr) was proven by X-ray absorption spectroscopy (XAS) at the Ru-edge, as well as cyclic voltammetry (see ESI† for details).

MIL-101-2@Ru and MIL-101-4@Ru were evaluated for catalytic water oxidation using Ce(iv) ammonium nitrate (CAN) as oxidant, and their performance compared to that of the homogeneous reference catalyst, [Ru(bda)(hep)(I-py)]. On the timescale of seconds, O_2_ evolution was monitored with a Clark-type electrode (Fig. 1), while longer timescale experiments were followed by gas chromatography (GC) (Table 1). The reaction conditions (pH ∼ 0.5, HNO_3_) were chosen based on similar reports of Ru(bda)-based homogeneous systems, and the total amount of Ru catalyst was kept identical in all experiments to allow for meaningful comparisons (*n*(Ru(bda)) = 0.034 μmol; *n*(CAN) = 2.5 μmol). This implies that the homogenous reference catalyst may have an advantage over the MOF-incorporated analogues, since in the homogenous case, all catalysts are accessible. Despite this potential disadvantage, we were pleased to find that MOF incorporation does not slow down the first few catalytic turnovers under these conditions (Fig. 1a). The initial rate of O_2_ evolution catalyzed by the homogeneous [Ru(bda)(hep)(I-py)] (40 μM s^–1^, corresponding to a TOF = 1.2 s^–1^) is almost identical to that of the heterogeneous MIL-101-2@Ru (36.7 μM s^–1^, TOF = 1.1 s^–1^). MIL-101-4@Ru with a higher catalyst loading exhibits a slightly slower initial rate (19.1 μM s^–1^, TOF = 0.5 s^–1^). In the absence of any Ru(bda), for example in MIL-101-2, no O_2_ can be detected.

**Fig. 1:**
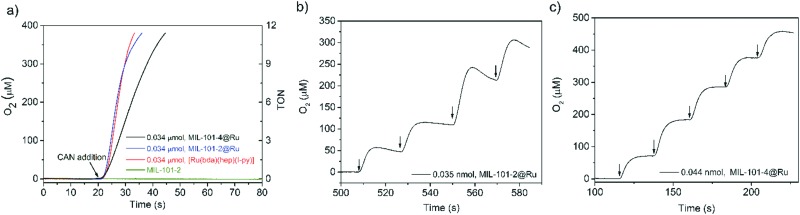
(a) Oxygen production plots using 50 μL CAN (0.05 M, 2.5 μmol) for homogeneous [Ru(bda)(hep)(I-py)], heterogeneous (MIL-101-2@Ru, MIL-101-4@Ru) catalyst and support (MIL-101-2) at pH ∼ 0.5. (b and c) O_2_ evolution of 0.035 μM MIL-101-2@Ru and 0.044 μM MIL-101-4@Ru in HNO_3_ (pH ∼ 0.5) by multiple additions of CAN (0.75 μM, 1.13 × 10^–3^ μmol). Arrows indicate the addition of CAN. Decrease in O_2_ concentrations after injection are due to O_2_ bubble formation.

**Table 1:** Ce(iv)-induced water oxidation for homogeneous and heterogeneous catalyst

Catalyst	Time (min)	Ru (μmol)	TON	TOF (s^–1^)
MIL-101-2@Ru^*a*^	5	0.054	550	1.9
MIL-101-2@Ru^*a*^	60	0.053	700	—
MIL-101-4@Ru^*b*^	60	0.22	1500	—
MIL-101-4@Ru^*b*^	5	0.22	1050	3.5
[Ru(bda)(hep)(I-py)]^*a*^	60	0.07	140	—
[Ru(bda)(pic)_2_]@SBA-16^4^	^*c*^	—	1200	4.5
MIL-101(Cr)	60	0	0	0

^*a*^0.5 M CAN (in 0.5 M HNO_3_, 1 mmol).

^*b*^0.67 M CAN (in 0.5 M HNO_3_, 2 mmol).

^*c*^TON was for 30 min and TOF was for first 2 min.^4^

The mechanistic implications of these experimental findings are complex. For example, assuming a statistical distribution of all Ru catalysts within the low-loading 101-2@Ru material would result in less than one catalyst per pore, and thus site-isolated species that could oxidize water only by a WNA mechanism. Conversely, by assuming a predominantly surface or near-surface functionalization, the local catalyst concentration may be sufficiently high to allow for the I2M mechanism. Complicating matters even more, reality most likely lies somewhere between these two extreme scenarios. Despite of these uncertainties, the kinetic data during the first few turnovers still allow for certain conclusions. The similar initial turnover frequencies (TOFs, amount of generated oxygen per second and total amount of Ru) of MIL-101-2@Ru and the homogenous reference complex suggest that the intrinsic activity of Ru(bda) is retained upon incorporation into the heterogeneous scaffold. As water oxidation catalysed by Ru(bda) operates by a I2M mechanism, the similar TOFs also imply that the Ru catalysts in MIL-101-2@Ru follow the same mechanism and that they are localized close to, or at the crystal surface. In such a case, also complicating factors arising from the mesoporous environment of MIL-101, *i.e.* diffusion of the oxidant and possibly counter ions, are no issues. In contrast, the higher loading MIL-101-4@Ru shows a lower initial formal TOF, which points towards a situation where the catalytic reaction is no longer limiting. These observations may point to only partial accessibility of the catalysts within the MOF or limiting CAN transport to all catalyst sites.^17^ Accessibility may be a more severe problem in the higher loading MIL-101-4@Ru for which the ICP/EDX analysis shows a larger proportion of catalysts in the interior of the crystals. While the surface-localized catalyst will still turn over similarly fast as in MIL-101-2@Ru, the formal TOFs of MIL-101-4@Ru will be smaller as they are normalized for the total amount of Ru present. As it is impossible to determine the ratio of Ru complexes that actually engage in catalysis, it is conceivable that the entire catalytic effect may arise from a small proportion of the catalysts at the surface of the crystals. In this case, the formal TOFs may considerably underestimate the turnover rates of the catalysts, and the formal TOFs would be merely lower estimates.

The stability and durability of the catalyst generally improves as a function of MIL-101(Cr) incorporation, as demonstrated by multiple addition experiments of several aliquots of CAN (1.13 × 10^–3^ μmol). As shown in Fig. 1b and c for 0.035 nmol MIL-101-2@Ru and 0.044 nmol MIL-101-4@Ru, respectively, repeated addition of oxidant re-establishes oxygen evolution at a similar rate as that during the previous addition. Over five cycles of injecting CAN into MOF suspensions, the oxygen production rate does not change significantly (Fig. S17, ESI†), which shows that the heterogeneous catalyst remains stable and active towards water oxidation. MIL-101 incorporation also prolongs the long-term stability of the Ru(bda) catalyst. For example, addition of a second aliquot of CAN to a suspension of MIL-101-2@Ru 24 hours after the first addition re-establishes O_2_ production at an appreciable rate (initially 39.0 μM s^–1^; after 24 h: 24.0 μM s^–1^), while the rate in the equivalent experiment with homogenous Ru(bda) decreases to 15% of the initial value. The degradation of the homogenous catalyst over 24 hours is also visible from a colour change of the solution from reddish yellow to light violet (Fig. S18 and S19, ESI†), while the MIL-101-2@Ru material appears unchanged to the eye and maintains its morphology as shown by SEM (Fig. S20, ESI†). This finding is consistent with a report by Das *et al.* who demonstrated unaltered PXRD spectra of MIL-101(Cr) materials after exposure to similarly high concentrations of CAN at pH = ∼1, and over similar timescales.^18^ In our hands, the PXRD patterns before and after water oxidation are less conclusive, potentially due to the presence of large amounts of decomposition products that arise from consumed oxidant (Fig. S21, ESI†).

The effect of the MIL-101 matrix to stabilize the structural integrity of the catalyst, and thus to promote higher turnover numbers (TONs) becomes apparent in water oxidation experiments on longer timescales and in the presence of a higher concentration of CAN (1–2 mmol). Under such conditions, the TONs of the MIL-101-incorporated Ru(bda) are considerably higher than those of the homogeneous reference compound (Table 1). After one hour, the TONs for MIL-101-2@Ru and MIL-101-4@Ru are 700 and 1500, respectively, compared to 140 of the homogenous reference catalyst [Ru(bda)(hep)(I-py)]. It turns out that most of the catalysis proceeds already in the first five minutes of the experiments when TONs of 550 and 1050 are measured for the two heterogeneous materials (Table 1). These TONs correspond to very respectable TOFs of 1.9 s^–1^ and 3.5 s^–1^, respectively.

These values compare favourably to most previously reported materials in which a molecular water oxidation catalyst has been incorporated into a heterogeneous support. For example, a biomimetic di-Mn catalyst encapsulated in MIL-101(Cr) displayed an initial TOF of 0.011 s^–1^,18*a* and several Cp*Ir(L)Cl (L = dibenzoate-substituted 2,2′-bipyridine) catalysts for two UiO-type MOFs showed TOF = 8.0 × 10^–3^ and 3.3 × 10^–3^ s^–1^.^17^ Additionally, a bipyridine-bridged periodic mesoporous organosilica (BPy-PMO) with Ir catalysts was reported with a TOF of 0.03–0.05 s^–1^.^19^ The TONs and TOFs are however lower by a factor of two compared to the Ru(bda)(pic)_2_@SMA-16 system (TON = 3300, TOF = 8.7 s^–1^) in which the bimolecular radical coupling mechanism is enforced.^4^

Remarkably, the TOFs in the presence of high oxidant concentrations are higher compared to the TOFs during the first few turnovers and lower [CAN] (see Fig. 1). This observation is consistent with the discussion above that the formal TOFs under lower [CAN] may actually be lower limits. Under higher oxidant concentrations, additional Ru centres that are localized further in the interior of the crystal may become accessible and participate in catalysis. This could be achieved either by diffusion of the oxidant onto MIL-101, or by hole hopping into the material as recently suggested by Morris and co-workers.^20^ A logical extension of this reasoning is that the higher loading MIL-101-4@Ru exhibits a higher TOF than MIL-101-2@Ru, as observed experimentally.

In summary, we have introduced a molecular water oxidation catalyst into MIL-101(Cr) by PSM methods, using the axial pyridine ligands as anchoring points.^21^ The MIL-101@Ru(bda) materials are active water oxidation catalysts, exceeding the TONs and TOFs of the homogenous reference [Ru(bda)(hep)(I-py)] by almost a factor of ten. While the exact location of all catalysts within the materials is admittedly difficult to determine, some generally applicable conclusions can be drawn from this study. In systems as complex as MIL-101@Ru(bda), the determined TOFs for catalytic water oxidation depend strongly on the concentration of the oxidant. At relatively low [oxidant], catalysts at the surface turn over sufficiently fast to keep up with the flux of oxidant. If these are the only catalysts in the material, the observed TOFs correspond to the actual kinetics of the catalyst. As soon as catalysts are in the crystal interior and are not catalytically active, the formally obtained TOFs are lower limits. By raising oxidant concentration, such catalysts can be accessed, either directly or through a hole hopping mechanism, and the observed TOFs increase markedly.

The authors would like to acknowledge the SuperXAS beam line at the Swiss Light Source (SLS) for access, and the Swedish Research Council, the Swedish Energy Agency, the Knut & Alice Wallenberg Foundation and the European Research Council (ERC-CoG2015-681895_MOFcat) for financial support, and DST-INSPIRE for an INSPIRE Faculty Fellowship to A. B. (DST/INSPIRE/04/2017/001072).

## Conflicts of interest

There are no conflicts to declare.

## Supplementary Material

Supplementary information
