# Supplementary material for: Formal water oxidation turnover frequencies from MIL-101(Cr) anchored Ru(bda) depend on oxidant concentration
Source: Chem Commun (Camb). 2018 Jun 12;54(56):7770–3. doi: 10.1039/c8cc02300j (PMC6040278; doi:10.1039/c8cc02300j)
Supplement: Supplementary file 1 [file CC-054-C8CC02300J-s001.pdf]

Electronic Supplementary Material (ESI) for ChemComm.
This journal is © The Royal Society of Chemistry 2018

Formal water oxidation turnover frequencies from MIL-101(Cr) anchored Ru(bda) depend on oxidant concentration

Asamanjoy Bhunia,[a, b] Ben A. Johnson,[a] Joanna Czapla-Masztafiak,[c] Jacinto Sá[a,d] and Sascha Ott*[a]

[a] *Department of Chemistry - Ångström Laboratory, Uppsala University Box 523, 75120 Uppsala (Sweden)*
[b] *Department of Chemistry, National Institute of Technology-Puducherry, Karaikal 609 609, India*
[c] *Institute of Nuclear Physics Polish Academy of Sciences, PL-31342 Krakow, Poland.*
[d] *Institute of Physical Chemistry, Polish Academy of Sciences, ul.Kasprzaka 44/52, 01-224 Warsaw, Poland.*

E-mail: sascha.ott@kemi.uu.se

Supporting Information (SI)

Experimental section

Materials

All chemicals and solvents were purchased from commercial suppliers (Sigma-Aldrich, TCI Europe, VWR, and Alfa Aesar chemical company) and used without further purification, unless stated otherwise. $[Ru^{II}(bda)(DMSO)_2]$ (H_2bda = 2,2'-bipyridine-6,6'-dicarboxylic acid; DMSO = dimethyl sulfoxide) was synthesized according to reported procedures.[1]

^{1}H-NMR spectra were measured using a JEOL 400 MHz spectrometer at 293 K. The chemical shifts given in ppm are internally referenced to the residual solvent signal. HPLC-MS data were obtained using a Dionex UltiMate 3000 system on a Phenomenex Gemini C18 column (150 × 3.0 mm, 5 µm) coupled to a Thermo LCQ Deca XP Max with electrospray ionization.

Powder X-ray diffraction patterns (PXRD) were obtained using a Simons D5000 Diffractometer (Cu Kα, λ = 0.15418 nm) at 45 kV and 40 mA, using a step size of 0.02°. Scanning electron microscopy (SEM) images were obtained using a Zeiss 1550 Schottky field-emission scanning electron microscope equipped with an InLens detector at 5 kV acceleration voltage. Samples were anchored to conductive carbon tape on a sample holder disk and coated using a Pd–Ir-sputter coater for 30 s.

N_2 adsorption isotherms were measured using a Micromeritics ASAP 2060. Materials were activated under dynamic vacuum (1×10^{-4} Pa) using a Micromeritic SmartVacPrep sample preparation unit. Helium gas was used for the determination of the cold and warm free space of the sample tubes. N_2 sorption isotherms were measured at 77 K (liquid nitrogen bath).

The ruthenium content in the MOF materials was determined by inductively coupled plasma atomic emission spectrometer (ICP-AES). It is equipped with a Spectro Cirros CCD (Kleve, Germany) detector with a modified-Lichte nebulizer and 14.0 L/min coolant Ar gas, 0.9 L/min plasma Ar gas and 0.9 L/min nebulizer Ar gas. 2-5 mg of MIL-101-2@Ru and MIL-101-4@Ru were digested in a mixture of conc. H_2SO_4:H_2O_2 (1 mL/0.5 mL) and heated in a Biotage (Uppsala, Sweden) SPX microwave reactor at 180 °C for 1 h. Afterwards, the solution became

clear. The resulting acidic solution was diluted to 5 mL with deionized H_2O and analyzed for Cr and Ru.

Chloromethylation of MIL-101(Cr) gave MIL-101-1 as described previously. MIL-101-2@Ru was synthesized directly by the reaction of MIL-101-1 and [Ru(bda)(hep)(I-py)] (hep = 4-(hydroxyethyl)pyridine)) in the presence of K_2CO_3 in DMF/acetone (1:1). MIL-101-2 was prepared as a reference material (without the catalytic Ru complex present) by etherification of MIL-101-1 with 4-(2-hydroxyethyl)pyridine in the presence of K_2CO_3 and 18-crown-6 ether, which afforded the pyridine-functionalized MIL-101-2. The latter was reacted with [Ru(bda)(DMSO)$_2$] in the presence of 4-iodopyridine (I-py) to confirm MIL-101-2@Ru could also be prepared via the reference material MIL-101-2 (see below for details). Both procedures towards MIL-101-2@Ru result in materials with comparable catalyst loadings. All studies described were done on the MIL-101-2@Ru material that was produced by the two-step procedure directly from MIL-101-1, and MIL-101-2 was used solely as a reference material without the attached catalytic unit. An alternative way to immobilize the Ru(bda) water oxidation catalyst into MIL-101(Cr) relies on an imine bond formation using PSM methods (see Scheme 1). Thus, amine-decorated MIL-101-3 was reacted with 4-pyridinecarboxaldehyde in a condensation reaction to afford MIL-101-4. The ruthenium catalyst was then anchored to the pyridine groups by treatment of MIL-101-4 with [Ru(bda)(DMSO)$_2$] and 4-iodopyridine to give MIL-101-4@Ru (see below for details).

Synthesis of MIL-101(Cr): MIL-101(Cr) was synthesized from chromium nitrate and benzene-1,4-dicarboxylic acid according to a literature procedure.[2] The as-synthesized MIL-101(Cr) were activated by washing with warm DMF (60°C), 1M HCl, H_2O, ethanol and dried in vacuo. The dried MIL-101(Cr) was used for chloromethylation.

Synthesis of MIL-101(Cr)-CH_2Cl (MIL-101-1): MIL-101-1 was synthesized as described previously.[3] A Schlenk flask equipped with a stir bar was charged with MIL-101(Cr) (200 mg) and nitromethane (14 mL) under Ar atmosphere. $AlCl_3·6H_2O$ (380 mg, 1.6 mmol) and metoxyacetyl chloride (80 mg, 0.74 mmol) were added sequentially to the vigorously stirred suspension. The mixture was heated at 100°C overnight. After cooling to room temperature, the

greenish chloromethylated product was collected by centrifugation. The product was activated using water (60 mL) at 60°C for 4 h, ethanol (60 mL) at 85°C for 4 h and after that with THF (60 mL) at 65°C for 4 h. Finally, the greenish powder was collected by centrifugation and dried under vacuum at 80°C.

Synthesis of MIL-101(Cr)-$CH_2OCH_2CH_2(C_5H_5N)$ (**MIL-101-2**): A Schlenk flask equipped with a stir bar was charged with MIL-101-1 (200 mg), K_2CO_3 (102 mg, 0.74 mmol) and 18-crown-6 (32 mg, 0.12 mmol) in acetone (30 mL) under Ar atmosphere. 4-pyridineethanol (91 mg, 0.74 mmol) was added with vigorous stirring. The suspension was reflux overnight and cooled down to room temperature. The product was washed with water (60 mL) at 60°C for 4 h, ethanol (60 mL) at 85°C for 4 h and after that with THF (60 mL) at 65°C for 4 h. Finally, the greenish powder was collected by centrifugation and dried under vacuum at 80°C.

Synthesis of MIL-101-2@Ru: A Schlenk flask equipped with a stir bar was charged with MIL-101-2 (100 mg) and [Ru(bda)$(DMSO)_2$] (80 mg, 0.16 mmol), methanol (20 mL), and DMSO (0.1 mL) under Ar atmosphere. The suspension was heated to 40°C for 5 min, and then 4-iodopyridine (20 mg, 0.097 mmol) was added. The reaction mixture was refluxed overnight and cooled down to room temperature. The greenish product was filtered and washed extensively with water (3×30 mL), TFE (4×10 mL), ethanol (3×30 mL), acetone (3×30 mL) and further purified by Soxhlet extraction for 12 h with $CHCl_3$. The final product was dried under vacuum at 80 °C.

Alternative Route for the Synthesis of MIL-101-2@Ru: 50 mg of 1-Cl, 65 mg of [Ru(bda)(4-iodopyridine)(hep)] (hep = 4-(hydroxyethyl)pyridine), and 80 mg of K_2CO_3 were suspended in DMF/acetone (5 mL/5 mL) under Ar atmosphere. The resulting reaction mixture was heat at 65°C overnight. The greenish product was filtered and washed extensively with water (3×30 mL), TFE (4×10 mL), ethanol (3×30 mL), acetone (3×30 mL), and further purified by Soxhlet extraction for 12 h with $CHCl_3$. The final product was dried under vacuum at 80 °C (40 mg).

Synthesis of [Ru(bda)(hep)(I-py)]: [Ru(bda)(hep)(I-py)] (hep = 4-(hydroxyethyl)pyridine) was prepared according to published methods.[4] A Schlenk flask was charged with [Ru(bda)$(DMSO)_2$] (100 mg, 0.2 mmol) and 4-iodopyridine (41 mg, 0.2 mmol), DMSO (0.1 mL), and methanol (20 mL) under Ar atmosphere. The resulting solution was heated to 40ºC for

5 min. 4-(hydroxyethyl)pyridine (24.7 mg, 0.2 mmol) was added and then refluxed overnight. The reaction mixture was cooled to room temperature and then evaporated under reduced pressure. The pure product was eluted by using methanol and dichloromethane mixture (1:15, v: v) as eluents. ^{1}H-NMR (DMSO-d_6) δ ppm: 8.70 (d, 2H), 7.88 (dd, 4H), 7.67 (d, 2H), 7.54 (d, 2H), 7.37 (d, 2H), 7.13 (d, 2H), 3.51(t, 2H), 2.59 (t, 2H). MS (ESI): calc for $[M+H]^+$, m/z = 672.82 (calc. = 672.95)

Synthesis of MIL-101-NO$_2$: 100 mg of MIL-101(Cr) was added to a mixture of conc. H_2SO_4 (7 mL) and conc. HNO_3 (5 mL) under stirring in an ice bath for 6 h. Then, the reaction mixture was poured into a beaker with 50 mL of crushed ice. The solid material was centrifuged off, washed with water (3×40 mL), ethanol ethanol (3×30 mL), acetone ethanol (3×30 mL) and then dried at room temperature.

Synthesis of MIL-101-3: 100 mg of MIL-101-NO$_2$ and 2 mg of $SnCl_2.2H_2O$ were suspended in EtOH (20 mL). The resulting suspension was heated at 75 °C overnight. The green powder was filtered and suspended again in 15 mL conc. hydrochloric acid. The greenish solid was filtered and then washed several times with water and finally with acetone.

Synthesis of MIL-101-4: 50 mg of MIL-101-3 and 0.1 mL of 4-pyridinecarboxaldehyde were suspended in 15 mL ethanol. The resulting suspension was heated to reflux overnight. Afterwards the solid was filtered, and washed with water several times and finally with acetone.

Synthesis of MIL-101-4@Ru: A Schlenk flask equipped with a stir bar was charged with MIL-101-4 (50 mg) and [Ru(bda)(DMSO)$_2$] (25 mg, 0.16 mmol), methanol (10 mL), and DMSO (0.1 mL) under Ar atmosphere. The suspension was heated to 40°C for 5 min, and then 4-iodopyridine (20 mg, 0.097 mmol) was added. The reaction mixture was refluxed overnight and cooled down to room temperature. The greenish product was filtered and washed extensively with water (3×30 mL), TFE (4×10 mL), ethanol (3×30 mL), acetone (3×30 mL), and further purified by Soxhlet extraction for 12 h with $CHCl_3$. The final product was dried under vacuum at 80 °C (42 mg).

Comparison of Cr:Ru ratio between ICP-AES and EDX

Sample: MIL-101-4@Ru	ICP AES	EDX
wt. ratio Cr:Ru	6.3	5.8
atomic ratio Cr:Ru	12.3	11.3

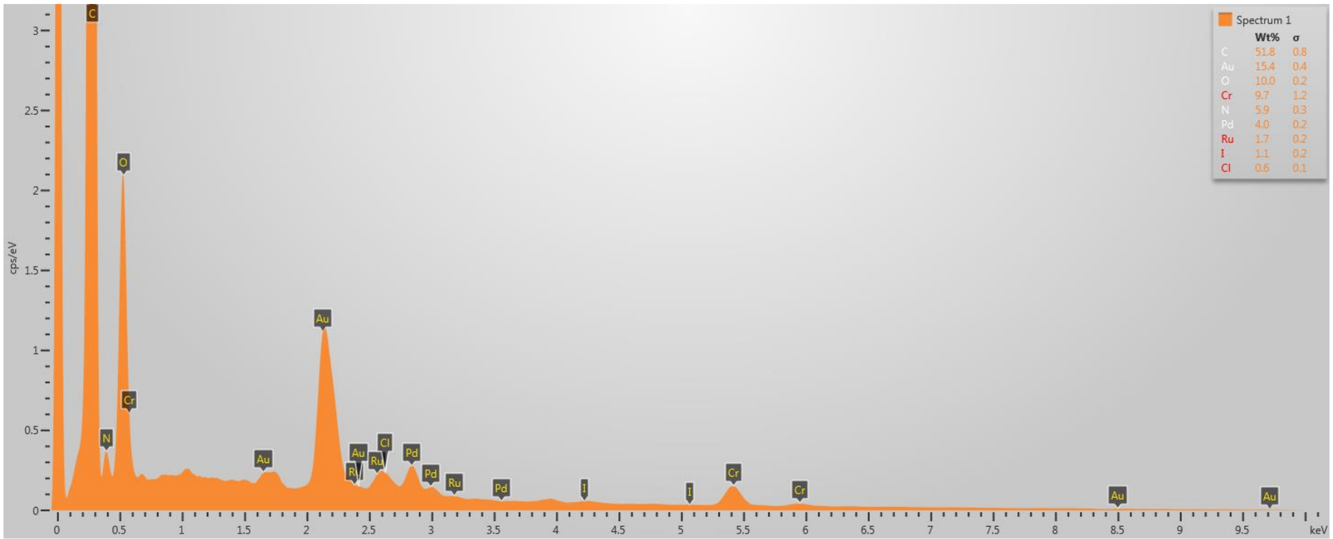

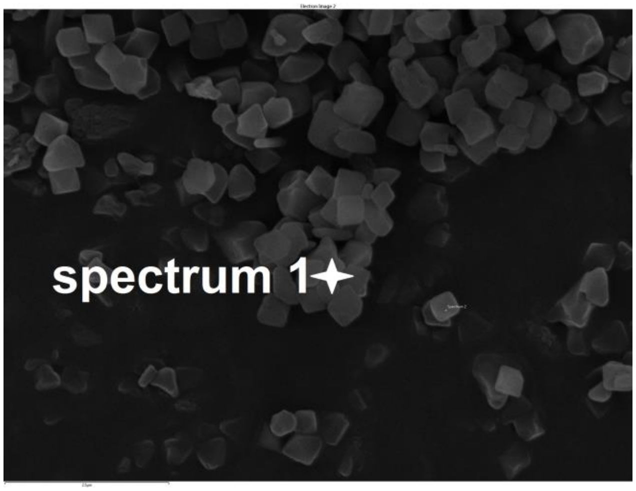

Figure S1. EDX spectrum of MIL-101-4@Ru (top) and SEM image of MIL-101-4@Ru (bottom).

Powder XRD:

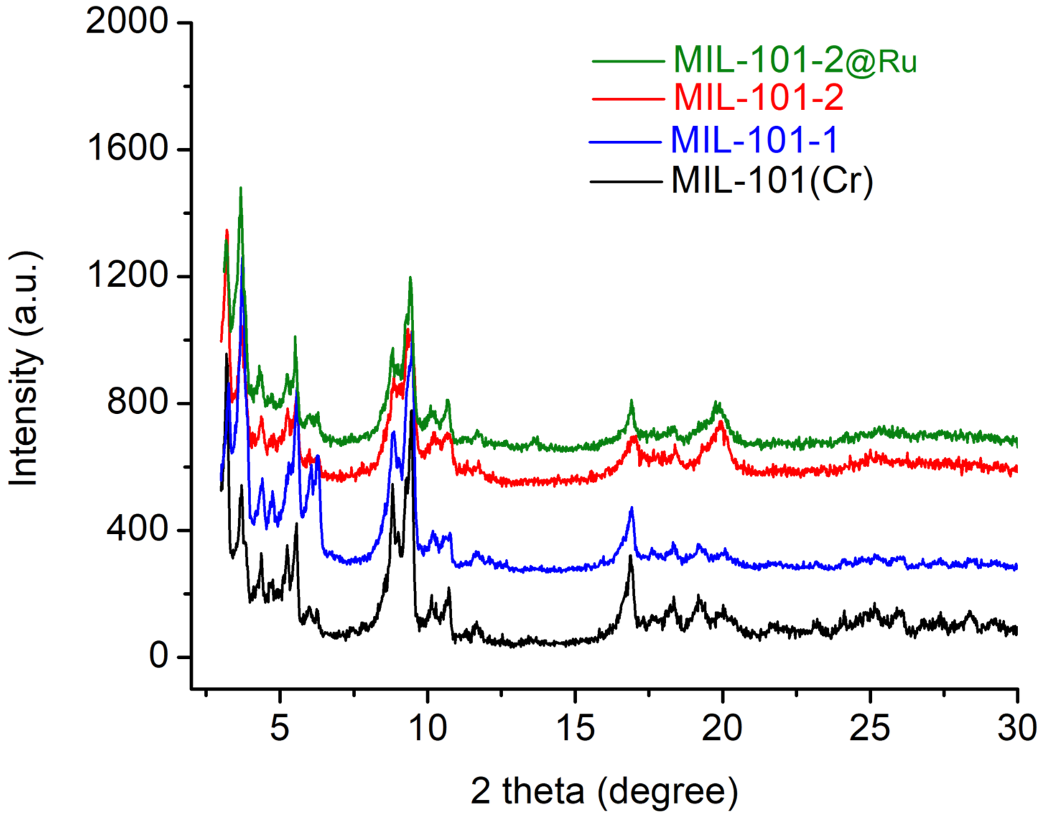

Figure S2. PXRD pattern of MIL-101(Cr) and its functionalized structures (MIL-101-1, MIL-101-2, and MIL-101-2@Ru).

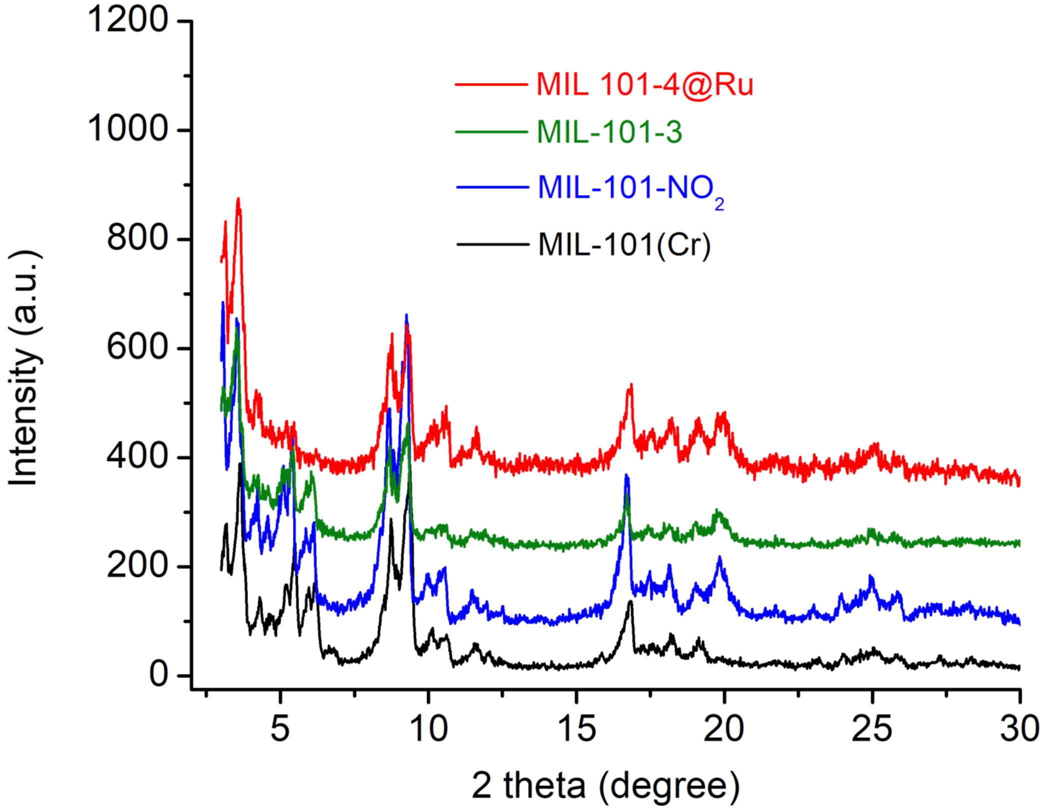

Figure S3. PXRD pattern of MIL-101(Cr) and its functionalized structures (MIL-101-NO_2, MIL-101-3, and MIL-101-4@Ru).

SEM images:

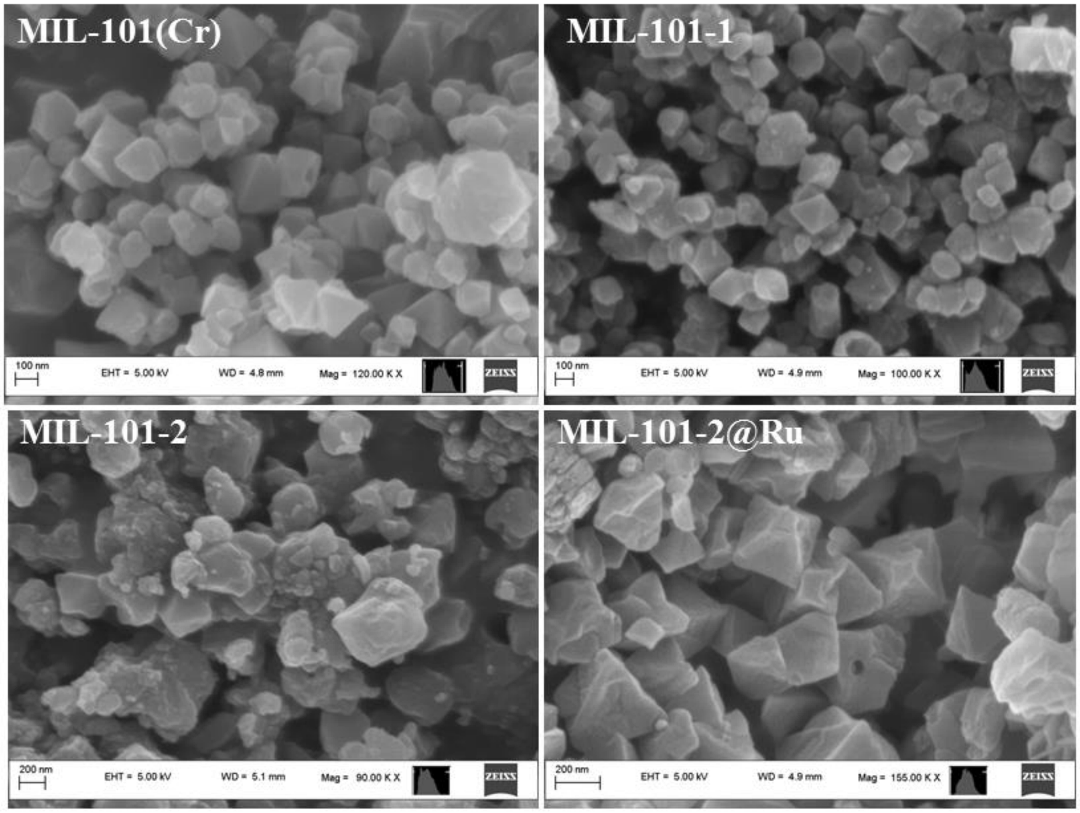

Figure S4. SEM images of MIL-101(Cr) and its functionalized structures (MIL-101-1, MIL-101-2, and MIL-101-2@Ru).

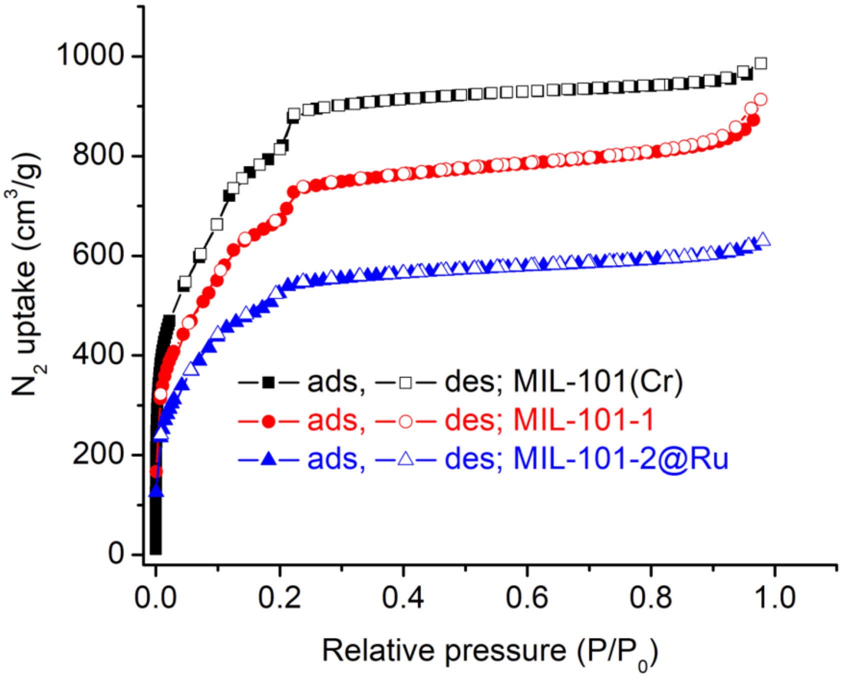

Figure S5. N_2 sorption isotherm at 77 K for MIL-101(Cr), MIL-101-1, and MIL-101-2@Ru.

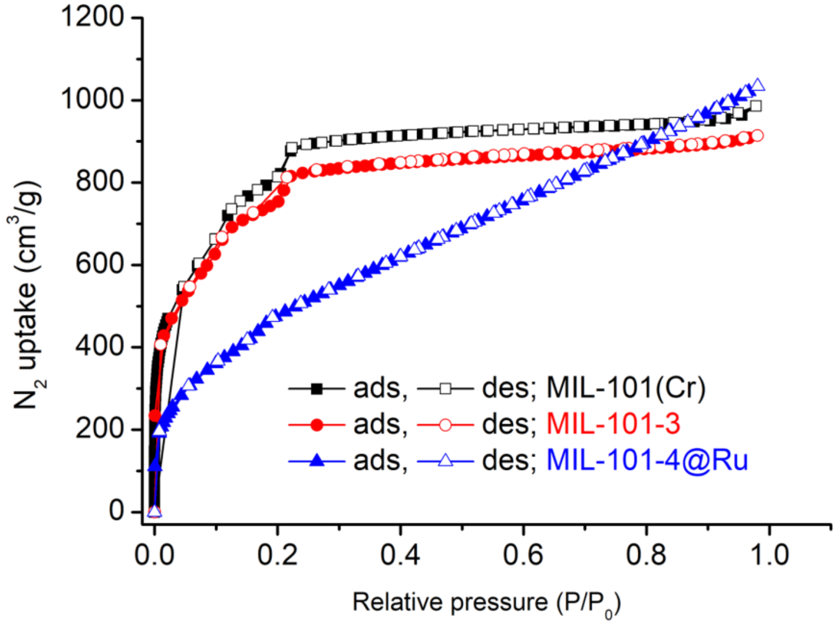

Figure S6. N_2 sorption isotherm at 77 K for 1, MIL-101-3 and MIL-101-4@Ru.

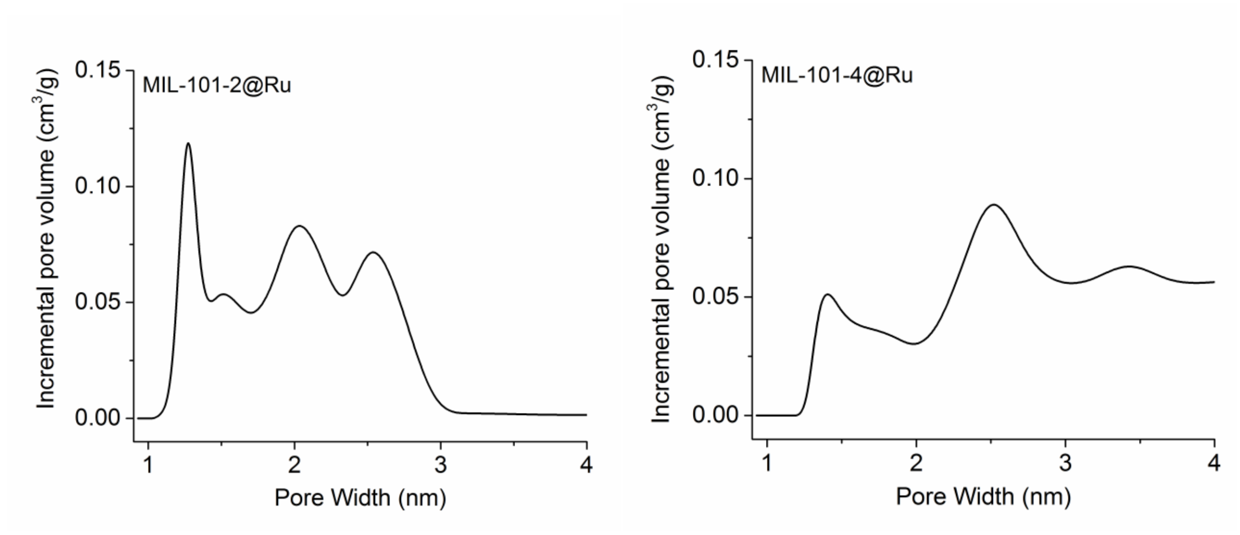

Figure S7. Pore size distribution of MIL-101-2@Ru (left) and MIL-101-4@Ru (right).

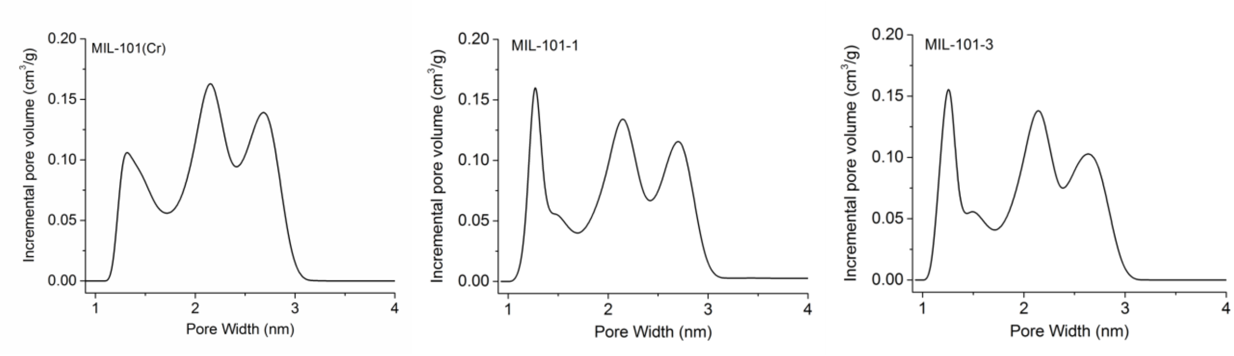

Figure S8. Pore size distribution of MIL-101(Cr) (left), MIL-101-1 (middle), and MIL-101-3 (right).

EXAFS analysis:

XAS experiments were performed on sample suspensions (20 mg of sample dispersed in 7 mL of DMSO). XAS spectra were recorded in fluorescence mode at the ruthenium K-edge (22.118 keV), by scanning the X-ray energy in 0.1 eV steps at the SuperXASbeamline of the Swiss Light Source (SLS), which operates with a ring current of approximately 400 mA in top-up mode. The polychromatic radiation from a superbend magnet, with a magnetic field of 2.9 T and critical energy of 11.9 keV, was monochromatized using a double crystal Si(311) monochromator. Samples were stirred during measurement to minimize radiation damage and the total acquisition time per sample was 30 min. XAS analysis provides information about the unoccupied electronic structure extracted from the X-ray Absorption Near Edge Structure (XANES) region and local structure information extracted from the Extended X-ray Absorption Fine Structure (EXAFS).

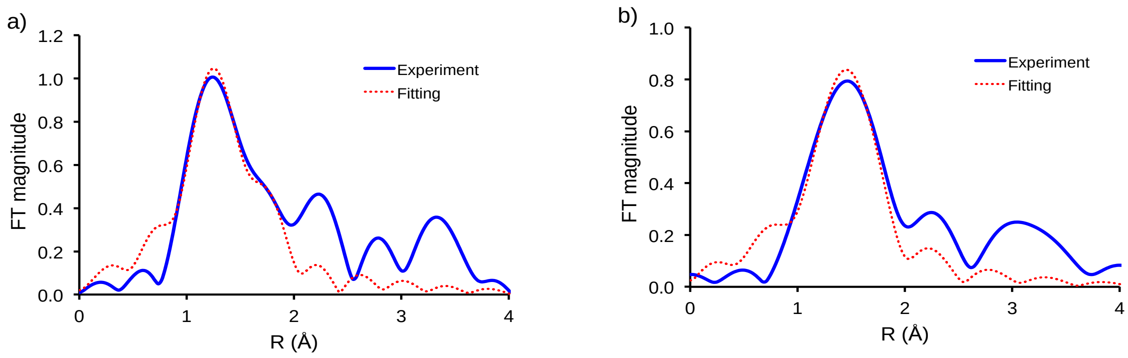

Figure S9. Fourier transform of Ru K-edge EXAFS for a) [Ru(bda)(hep)(I-py)] and b) MIL-101-2@Ru.

The EXAFS analysis revealed a significant change in the local geometric structure of the complex upon functionalization, which is clearly depicted by the Fourier analysis of the signal (Figure S9). EXAFS spectra were fitted with Artemis program (part of Demeter package).[5] The fitting of Ru(bda) yielded three types of nearest neighbors, namely two Ru—N at 1.865 Å, two Ru—N at 1.961 Å and two Ru—O at 2.120 Å, consistent with the expected values from reference structure (1.950, 2.077 and 2.216 Å, respectively). Upon functionalization, the best fitting (with R-factor of 0.017) was attained with only one type of neighbor, namely Ru—N/O at 2.074 Å. Note that due to the proximity in Z-number EXAFS is not able to distinguish between N and O if they are located at the same distance. Thus, functionalization resulted in an increase in the molecule symmetry comparatively to the pristine complex. Since there was not a

significant change in the XANES, one expects that these geometric structural changes to affect only the complex HOMO structure. However, one should know that the EXAFS signal's characteristically low signal-to-noise affects data fitting confidence. Therefore, the structural changes observed might not surmount to significant electronic structure changes, which dominate the catalytic process.

XANES analysis:

Figure S10 shows the XAS spectra of MIL-101-2@Ru and its molecular reference [Ru(bda)(hep)(I-py)]. There is virtually no difference in the XANES (X-ray absorption near-edge structure) region, both in white line position and intensity, suggesting that the molecular and MOF-incorporated complex have identical unoccupied electronic structures. The white line inflection point for both samples was located at 22.125 keV, characteristic of ruthenium in the oxidation state +II.[6]

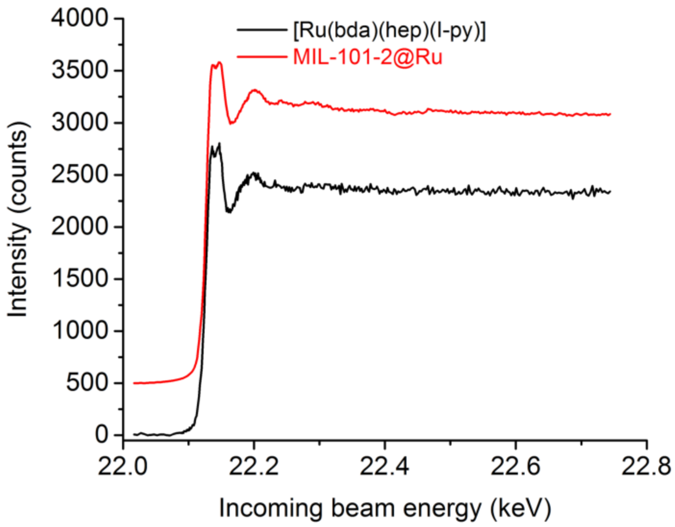

Figure S10. Ru K-edge XAS spectra for Ru(bda)(hep)(I-py) and MIL-101-2@Ru.

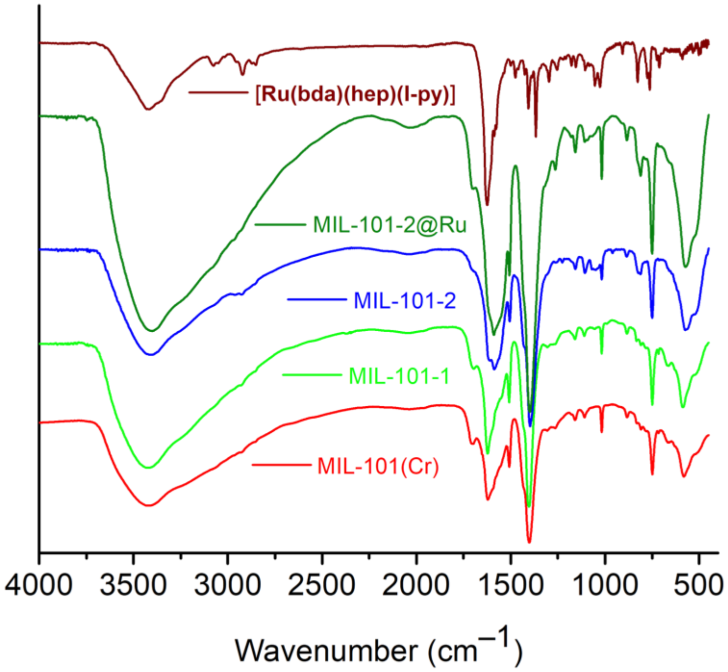

Figure S11. FT-IR spectrum of MIL-101 and its functionalized structures (MIL-101-1, MIL-101-2, and MIL-101-2@Ru).

Electrochemistry:

Cyclic voltammetry (CV) was performed using a one-compartment, three-electrode configuration connected to an Autolab PGSTAT100 potentiostat controlled with GPES 4.9 software (EcoChemie). The electrode setup included an auxiliary glassy carbon disc (0.071 cm^2) working electrode, which was used to monitor the solution between scans, a platinum rod counter electrode, and a Ag/AgCl aqueous reference electrode (sat. $KCl_{(aq)}$, 0.198 V vs. NHE). The counter electrode and the reference electrode were separated from the working electrode by salt bridges with glass frits filled with supporting electrolyte. MIL-101-2@Ru and MIL-101-2 films were assembled by drop casting 20 µL of a suspension of 0.4 mL EtOH with 5% v/v Nafion containing 2 mg of the MOF sample onto FTO and drying in ambient air overnight. The films were measured in aqueous solutions as the working electrode, using 0.1M borate buffer with $KNO_{3\ (aq)}$ to adjust the ionic strength (pH = 8.5, I = 1M) as a supporting electrolyte. The potential was converted to NHE (normal hydrogen electrode) by E(NHE) = E(Ag/AgCl) + 0.198V

In support of the XAS studies, cyclic voltammograms (CVs) feature a reversible wave at the expected potential for a molecularly well-defined and structurally intact Ru(dba). In the anodic scan, the $Ru^{III/II}$ oxidation is observed as a reversible redox couple at 0.53 V *vs.* Ag/AgCl (0.73 vs. NHE), while any such feature is absent in MIL-101-2 (Figure S12). The potential is very close to that of $[Ru(bda)L_2]$ electropolymerized on glassy carbon electrodes which showed the $Ru^{III/II}$ couple at 0.69 V vs. NHE.[7] The non-zero peak separation (ΔE_p) and linear relationship between the peak current i_{pa} and $v^{1/2}$ for the $Ru^{III/II}$ couple in MIL-101-2@Ru is indicative of a diffusion limited process (Figure S13-S15), which has been demonstrated in numerous other MOF-catalyst systems.[8]

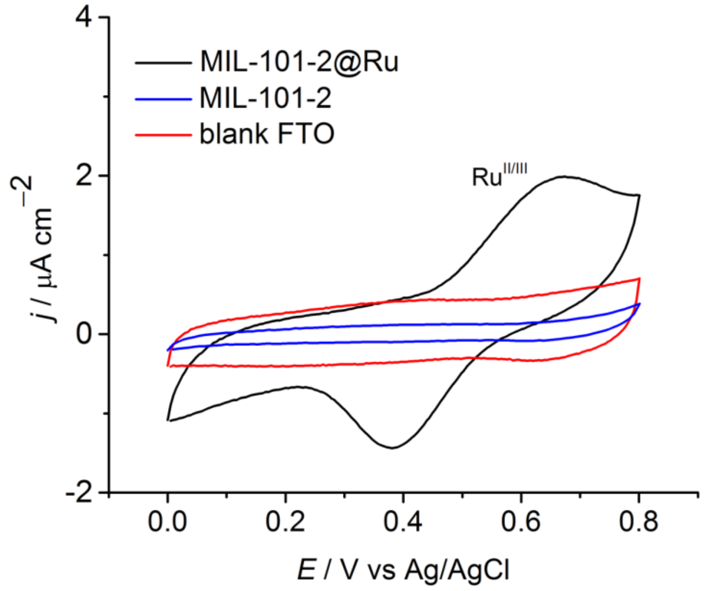

Figure S12. CVs of blank FTO, MIL-101-2 and MIL-101-2@Ru deposited on FTO at a scan rate of 100 mV s^{-1} in borate buffer / KNO_3 (pH = 8.5, I = 1 M).

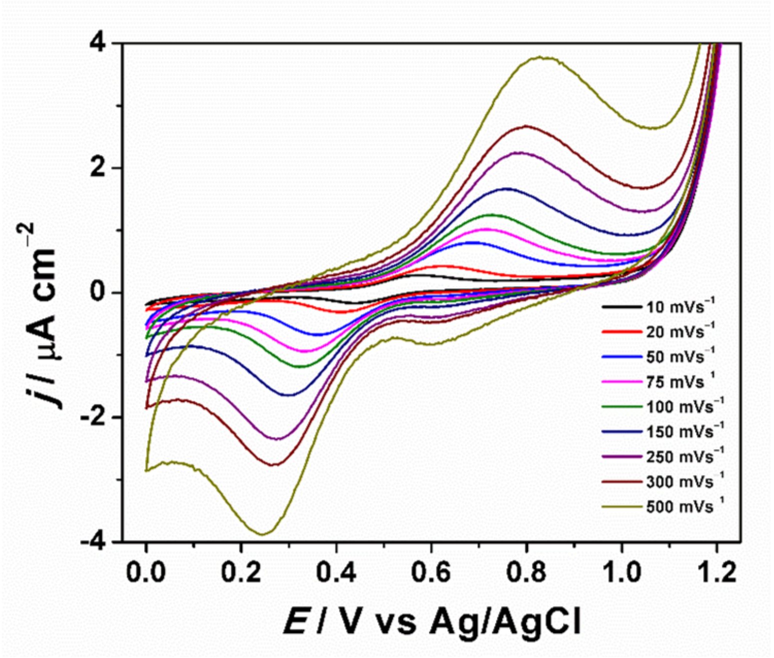

Figure S13. CVs of MIL-101-2@Ru deposited on FTO at scan rates from 10 mV s^{-1} – 500 mV s^{-1} in borate buffer / $KNO_{3\ (aq)}$ (pH = 8.5, I = 1 M).

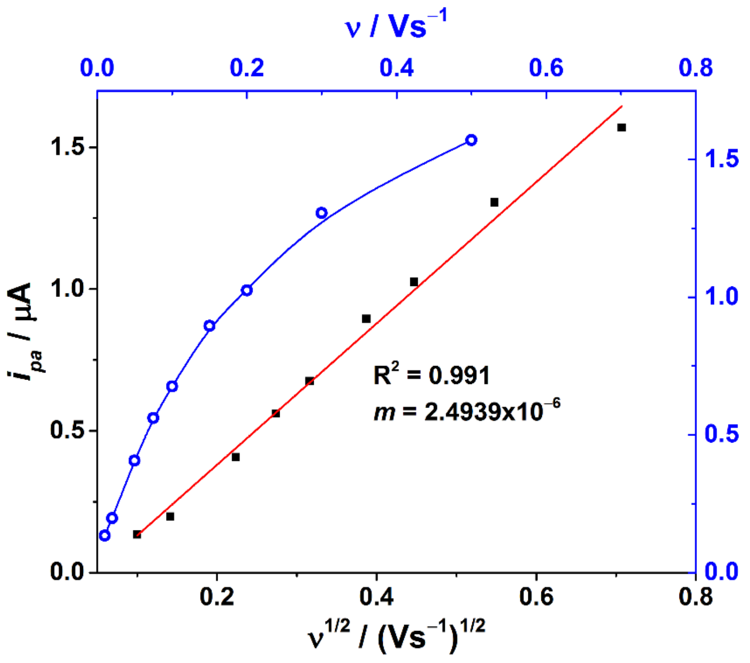

Figure S14. Plot of peak current, i_{pa} vs. $v^{1/2}$ (black) and i_{pa} vs. v (blue), showing a linear dependence of i_{pa} on $v^{1/2}$, which indicates a diffusion controlled process for MIL-101-2@Ru deposited on FTO at scan rates from 10 mV s^{-1} – 500 mV s^{-1} in borate buffer / $KNO_{3\ (aq)}$ (pH = 8.5, I = 1 M).

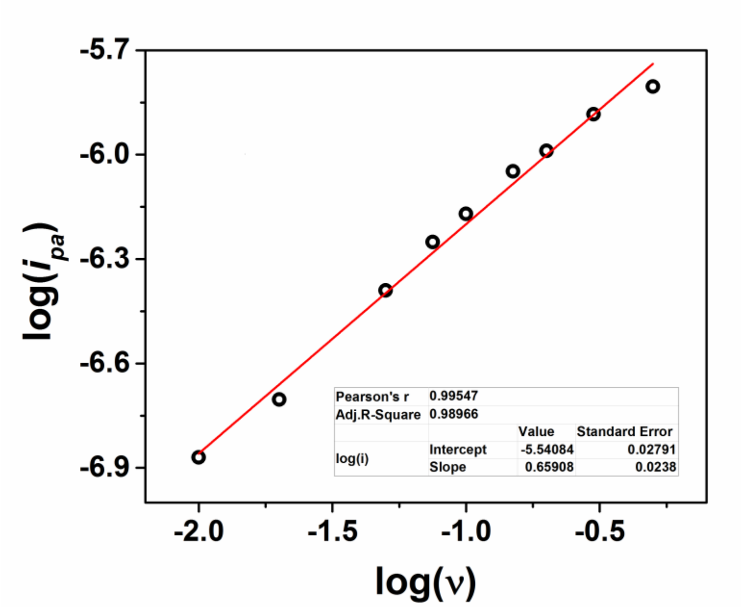

Figure S15. Plot of log(i_{pa}) vs. log(v) from CVs of MIL-101-2@Ru deposited on FTO in borate buffer / $KNO_{3\ (aq)}$ (pH = 8.5, I = 1 M) at scan rate from 10 mV s^{-1} to 500 mV s^{-1}. Inset shows a linear fit to this data and the slope = 0.65, indicating $i_{pa} \propto v^{1/2}$.

Control reaction of MIL-101(Cr) and [Ru(bda)(4-iodopyridine)(4-pyridineethanol)]:

In a 50 mL Schlenk flask 50 mg MIL-101(Cr), 50 mg (0.1 mmol) [Ru(bda)(DMSO)$_2$] and 20.5 mg (0.1 mmol) 4-iodopyridine were suspended in 0.05 mL DMSO and 20 mL methanol with stirring under Ar atmosphere. The obtained solution was heated to 40ºC for 5 min. 4-pyridineethanol (12 mg, 0.097 mmol) was added and then reflux overnight. The greenish solid was collected by filtration, and then washed extensively with water (3×30 mL), TFE (4×10 mL), ethanol (3×30 mL), and acetone (3×30 mL). It was then dried under vacuum at 100°C. The amount of adsorbed Ru complex was checked after microwave digestion (con. H_2SO_4/H_2O_2 (1mL/0.5 mL at 180°C for 1 h) via ICP-AES. However, we did not found significant amount of ruthenium after the digestion of the sample.

Oxygen evolution experiments:

A standard Clark-type oxygraph electrode (Hansatech Instruments), which is separated from the sample solution by a Teflon membrane, was used to check the O_2 production. For all experiments, the cell was thermostatted at 20 °C. The signal was recorded for the entire duration of the experiment at 0.1 s intervals using the Oxygraph+ software (Hansatech Instruments). The signal was calibrated using air saturated aqueous solutions ([O_2] = 271 μM, T = 20 °C).

ICP-AES and Loading of Ru(bda) in MIL-101(Cr):

MIL-101-4@Ru

The MOF sample (2.2 mg) was dissolved in 1 mL H_2SO_4/H_2O_2 (1:0.5) and then diluted to 5 mL for ICP-AES analysis. The concentrations of ruthenium and chromium in MIL-101-4@Ru after digestion in con. H_2SO_4/H_2O_2 were found to be 4.5 and 28.4 μg/mL respectively.

$$\frac{4.5\,\mu g\ Ru}{28.4\,\mu g\ Cr}\times\frac{1}{101.07\,mol\ Ru}\times\frac{51.996\,mol\ Cr}{1}=0.0815\ \frac{mol\ Ru}{mol\ Cr}$$

The reported chemical formula for MIL-101(Cr) is [$Cr_3(\mu_3\text{-}O)(OH)\text{-}(BDC)_3(H_2O)_2$][6]. Since Cr and BDC are in a 1:1 ratio, the percentage of functionalized linkers is 0.0815/1 = 8.15%.

The loading of Ru(bda) in MIL-101-4@Ru in μmol mg^{-1} was also determined:

$$4.5\ \frac{\mu g\ Ru}{mL} \times\ 5\ mL\ \times \frac{1 \mu g}{101.07\ \mu mol\ Ru} = 0.22\ \mu mol\ Ru \quad \frac{0.22\ \mu mol\ Ru}{2.2\ mg\ MOF} = 0.101 \frac{\mu mol\ Ru}{mg\ MOF}$$

MIL-101-2@Ru

The MOF sample (1.86 mg) was dissolved in 1 mL H_2SO_4/H_2O_2 (1:0.5) and then diluted to 5 mL for ICP-AES analysis. The concentrations of ruthenium and chromium in MIL-101-2@Ru after digestion in con. H_2SO_4/H_2O_2 were found to be 1.99 and 67.2 μg/mL respectively.

$$\frac{1.99\ \mu g\ Ru}{67.2\ \mu g\ Cr} \times \frac{1}{101.07\ mol\ Ru} \times \frac{51.996\ mol\ Cr}{1} = 0.0152\ \frac{mol\ Ru}{mol\ Cr}$$

The reported chemical formula for MIL-101(Cr) is $[Cr_3(\mu_3\text{-}O)(OH)\text{-}(BDC)_3(H_2O)_2]$[6]. Since Cr and BDC are in a 1:1 ratio, the percentage of functionalized linkers is 0.0152/1 = 1.52%.

The loading of Ru(bda) in MIL-101-2@Ru in μmol mg^{-1} was also determined:

$$1.99\ \frac{\mu g\ Ru}{mL} \times 5\ mL \times \frac{1 \mu g}{101.07\ \mu mol\ Ru} = 0.098\ \mu mol\ Ru \quad \frac{0.098\ \mu mol\ Ru}{1.86\ mg\ MOF} = 0.0527 \frac{\mu mol\ Ru}{mg\ MOF}$$

Calculation of TON and TOF:

TON and TOF were calculated by monitoring the amount of oxygen via GC in the headspace of a sealed vial containing a suspension of the MOF sample in 0.5M HNO_3 (1 mL) before and after addition of a CAN solution. Any dissolved oxygen in the solution was not considered. A calibration curve (Figure S16) was constructed by injecting known volumes (5 – 50 µL) of oxygen.

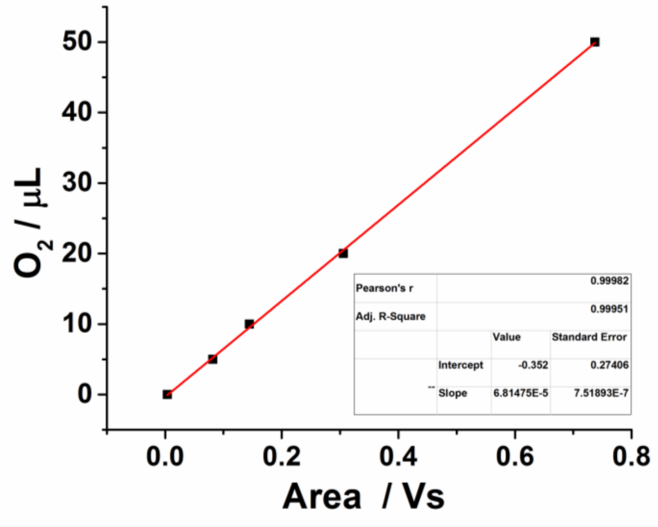

Figure S16. Calibration curve was constructed by injecting known volumes (5 – 50 µL) of oxygen.

In each experiment, the MOF suspension was added to the vial, which was sealed and degassed by passing Ar through the solution for 30 min. The total headspace volume was 8.9 mL or 28.7 mL depending on the size of the reaction vial for each experiment. Then, 100 µL of the headspace was injected into the GC prior to the addition of CAN to establish a baseline oxygen level. Subsequently, the CAN solution was added and the resulting suspension stirred. At different time intervals, the headspace was sampled by injecting 100 µL into the GC. The final amount of oxygen produced was then obtained using the calibration curve and back-calculating the concentration of oxygen in the headspace, $[O_2]$ (v/v), correcting for the baseline oxygen levels. A sample calculation for TON and TOF is given below:

For [Ru(bda)(hep)(I-py)],

t (min)	O_2 Peak Area (Vs)	Volume O_2 injected (μL)
0	16206	1.104
60	54429	3.709

***t* = 0 min**

$$[O_2] = \frac{1.104\ \mu L\ injected}{100\ \mu L} = 0.01104$$

$$V_{O_2}(headspace) = 0.01104\ [O_2]\ \times\ 8.9\ mL\ headspace = 0.09829\ mL$$

***t* = 60 min**

$$[O_2] = \frac{3.709\ \mu L\ injected}{100\ \mu L} = 0.03709$$

$$V_{O_2}(headspace) = 0.03709\ [O_2]\ \times\ 8.9\ mL\ headspace = 0.3301\ mL$$

$$\Delta V_{O_2}\ (headspace) = V_{O_2}^{t=60} - V_{O_2}^{t=0} = 0.3301\ mL - 0.09829\ mL = 0.2318\ mL$$

The volume of oxygen was converted to moles using PV = nRT where R = 8.34144 L kPa K^{-1} mol^{-1}, T = 298.15 K, P = 101.325 kPa and V = $2.318x10^{-4}$ L.

$$\Delta O_2\ (headspace) = 9.48\ \mu mol$$

Finally, the TON was calculated by,

$$TON = \frac{\Delta O_2\ (headspace)}{\mu mol\ Ru\ catalyst}$$

$$TON = \frac{9.48\ \mu mol\ O_2}{0.0694 \mu mol\ Ru\ catalyst} = 140$$

For MIL-101-4@Ru,

t (min)	O_2 Peak Area (Vs)	Volume O_2 injected (μL)
0	2018	0.14
5	416914	28.4

***t* = 0 min**

$$[O_2] = \frac{0.14\ \mu L\ injected}{100\ \mu L} = 0.0014$$

$V_{O_2}(headspace) = 0.0014\ [O_2]\ \times\ 28.7\ mL\ headspace = 0.04\ mL$

***t* = 60 min**

$$[O_2]\ =\ \frac{28.4\ \mu L\ injected}{100\ \mu L} = 0.284$$

$V_{O_2}(headspace) =\ 0.284\ [O_2]\ \times\ 28.7\ mL\ headspace = 8.15\ mL$

$\Delta V_{O_2}\ (headspace) =\ V_{O_2}^{t=60}\ -\ V_{O_2}^{t=0} = 8.15\ mL - 0.04\ \ mL = 8.11\ mL$

The volume of oxygen was converted to moles using PV = nRT where R = 8.34144 L kPa K^{-1} mol^{-1}, T = 298.15 K, P = 101.325 kPa and V = $6.198x10^{-3}$ L.

$\Delta O_2\ (headspace)\ = 332\ \mu mol$

$$TON = \frac{332\ \mu mol\ O_2}{0.216\ \mu mol\ Ru\ catalyst} = 1500$$

The amount of Ru catalyst was determined by the catalyst loading (µmol mg^{-1}) in [MIL-101-4@Ru as described above, which is 0.101 µmol mg^{-1}.

The TOF was obtained from the data taken after 5 min by,

$$TOF = \frac{TON}{\Delta t} = \frac{1050}{5\ min} \times \frac{1\ min}{60\ s} = 3.5\ s^{-1}$$

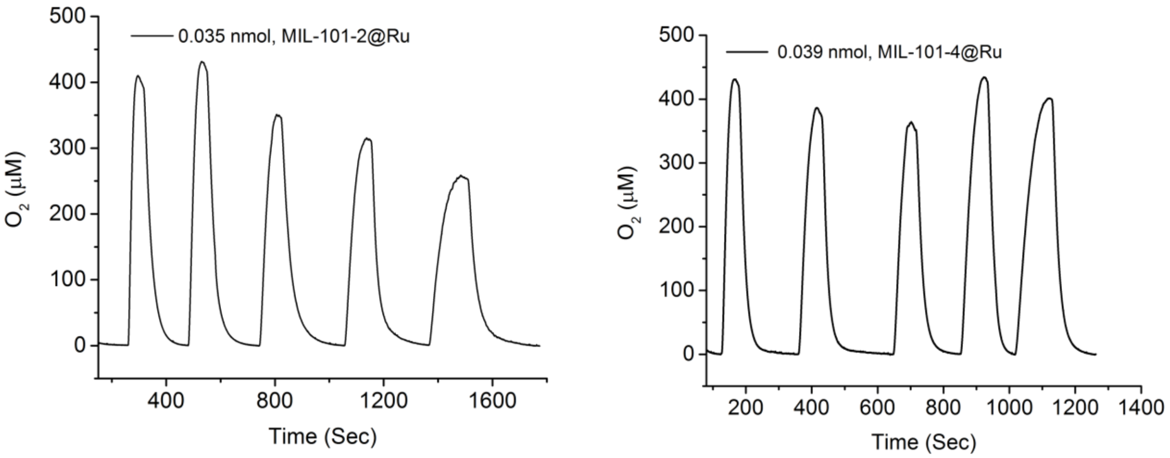

Figure S17. Water oxidation (Top: MIL-101-2@Ru; Bottom: MIL-101-4@Ru under repeated addition of CAN in a Clark-type electrode.

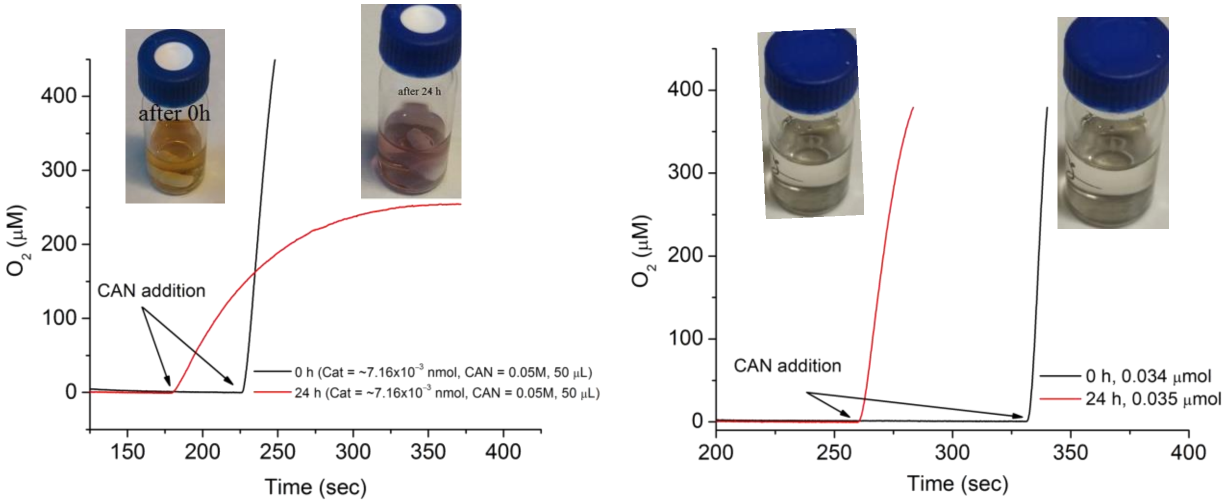

Figure S18. Left: O_2 evolution of homogeneous catalyst [Ru(bda)(hep)(I-py)] at pH ~0.5 during 0 h and 24 h, where the rate of O_2 evolution decreases after 24 h. Right: O_2 evolution of MIL-101-2@Ru at pH ~0.5 during 0 h and 24 h, where the rate of O_2 evolution is almost the same after 24 h.

Recyclability and reusability test:

15.154 mg of MIL-101-2@Ru was suspended in 1 mL HNO_3 solution (0.5 M) and purged with argon for 15-30 min in a Microwave reaction vial. A freshly prepared CAN (0.174 mg in 1 mL of 0.5 M HNO_3) solution outgassed with argon for 1 h was injected into the Microwave reaction vial. After 10 min, 100 μL gas in the head space was injected in the GC in order to quantify the evolved O_2. The suspension was then centrifuged to recover the catalyst, which was washed with H_2O and used in a subsequent run under identical conditions (0.174 mg CAN in 1mL of 0.5 M HNO_3). O_2 evolution was detected by GC for three consecutive runs, demonstrating the recyclability of this material.

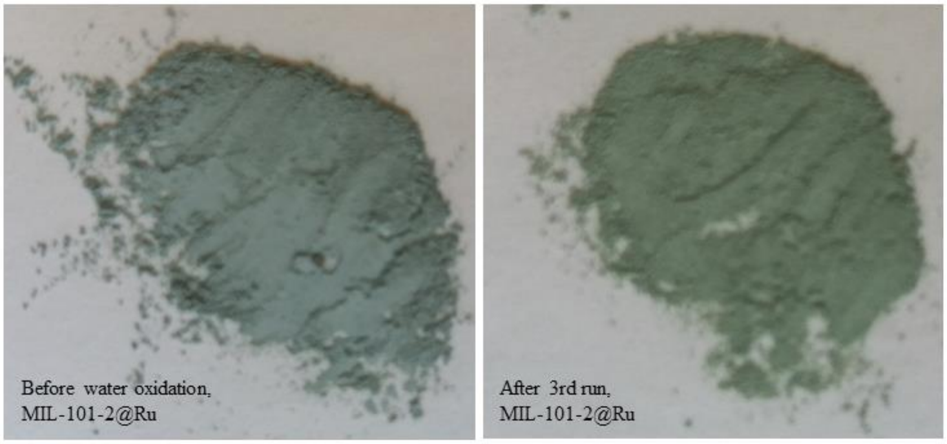

Figure S19. Photograph of MIL-101-2@Ru before (left side) and after (right side) catalysis.

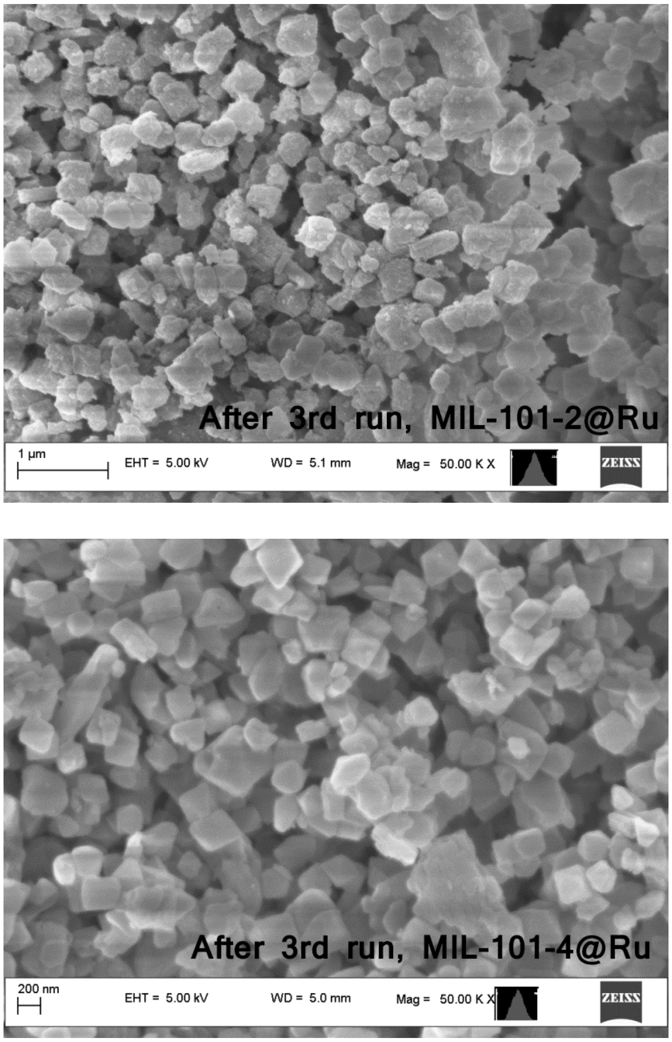

Figure S20. SEM image of MIL-101-2@Ru (top) and MIL-101-4@Ru (bottom) after catalysis.

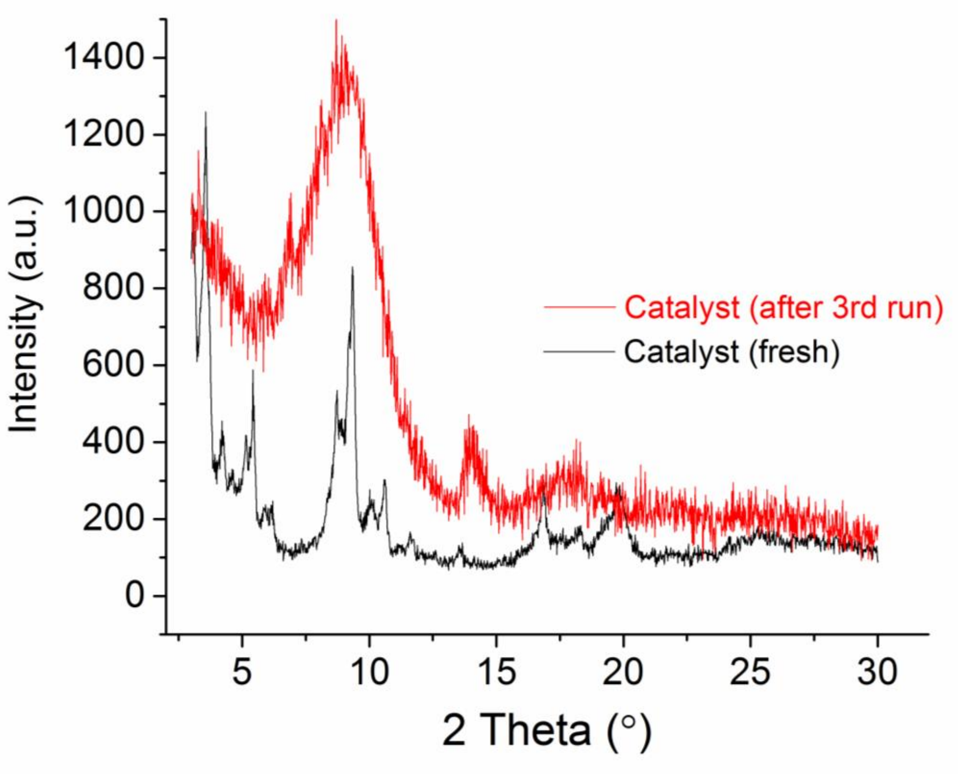

Figure S21. PXRD of MIL-101-2@Ru before and after catalysis.

Analysis of supernatant solution after catalysis:

The supernatant after catalysis with MIL-101-4@Ru was analyzed by ICP-AES and subject to a second addition of CAN. To MIL-101-4@Ru (2.13 mg, 0.22 μmol Ru) was added 0.67 M CAN under identical condition as shown in Table 1 (0.5 M HNO_3, 1 mL). After 5 min, the oxygen evolved was measured by GC. The suspension was centrifuged and filtered, and the supernatant was collected. A second addition CAN (0.67 M) to the supernatant (1 mL) revealed negligible oxygen evolution levels compared to the as-prepared catalytic materials. ICP-AES also showed amounts of leaching of Ru into the solution during catalysis. However, this does not lead to the formation of an active WOC species in solution, considering the low amount of oxygen produced during a second CAN addition to the supernatant. We attribute the presence of Cr in the supernatant to presence of small MIL-101-4@Ru crystallites (less than 200 nm) that are impossible to remove by centrifugation.

WOC	Time (min)	Ru (μmol Ru)	O2 evolved (μmol)	TON
**MIL-101-4@Ru**	5	0.22	240	1100
**supernatant**	5	--	6.5	--

ICP-AES	Total Cr (μmol)	Total Ru (μmol)	% Ru leached
**supernatant**	**0.78**	**0.16**	**44%**

References:

[1] Y. Gao, X. Ding, J. Liu, L. Wang, Z. Lu, L. Li and L. Sun, *J. Am. Chem. Soc.* **2013**, *135*, 4219–4222.
[2] D. Jiang, L. L. Keenan, A. D. Burrows and K. J. Edler, *Chem. Commun.* **2012**, *48*, 12053-12055.

[3] M. G. Goesten, K. B. S. S. Gupta, E. V. Ramos-Fernandez, H. Khajavi, J. Gascon, F. Kapteijn, *CrystEngComm*, **2012**, *14*, 4109.
[4] L. Wang, K. Fan, Q. Daniel, L. Duan, F. Li, B. Philippe, H. Rensmo, H. Chen, J. Sun, L. Sun, *Chem. Commun.* **2015**, *51*, 7883-7886.

[5] B. Ravel, M. Newville, *J.Synchr.Radiat.* **2005**, *12*, 537–541.

[6] a) F. R. García-García, M. Fernándes-García, M. A. Newton, I. Rodrígues-Ramos, A. Guerrero-Ruiz, ChemCatChem 2013, 5, 2446-2452; b) C. J. Pellicione, E. V. Timofeeva, J. P Katsoudas, C. U. Segre, *J. Phys. Chem. C* **2013**, *117*, 18904-18912.

[7]L. Wang, K. Fan, Q. Daniel, L. Duan, F. Li, B. Philippe, H. Rensmo, H. Chen, J. Sun, L. Sun, Chem. Commun. 2015, 51, 7883-7886.

[8] a) I. Hod, M. D. Sampson, P. Deria, C. P. Kubiak, O. K. Farha, J. T. Hupp, *ACS Catal.* **2015**, *5*, 6302-6309; b) S. R. Ahrenholtz, C. C. Epley, A. J. Morris, *J. Am. Chem. Soc.* **2014**, *136*, 2464-2472.
